# Involuntary Stools—Phosphorus

**Published:** 1884-10

**Authors:** J. T. Kent

**Affiliations:** St. Louis


### INVOLUNTARY STOOLS—PHOSPHORUS.

PROF. J. T. KENT, A. M., M. D., ST. LOUIS.

A lad eight years of age had been treated allopathically for five years, without any benefit, for losing his urine and stools in his pants. His mother informs me that she has often whipped him, thinking that he could prevent it. When she would go for the whip he would seem to be worse, and immediately soil himself from the fright. The stool passes without any warning, or it comes on too soon for him to accommodate himself. It seldom occurs at night or in the forenoon, but in the afternoon he passes several stools and always passes urine with stools. He takes cold easily, and when he gets a cold he has a high fever and delirium, and sometimes becomes croupy. The color of the stool is brown and the smell is very offensive. Urine stains the linen dark brown and has a strong smell. For the choice of remedy:

Involuntary stools and urine—Acon., Ars., *Bell.*, Bry., Calc., Camph., Carbo v., China, Cina, Colch., Con., Dig., *Hyos.*, *Laur.*, Mosch., Mur-rac *Nat. m.*, *Phos.*, Phos. acid, Puls., Rhus, Secale, Sulph., Verat.

The afternoon aggravation is characteristic of Bell.

Every time the child takes cold he has a high fever, and delirium is also characteristic of Bell. The general features of the case being covered by Bell., it was given two powders 4$^{m}$, with instructions to watch and make a fuller report of his symptoms.

One month after taking the medicine, the mother writes:

"My son is very much better, but not entirely cured. He has had only two involuntary stools since taking the medicine, both between 12 M. and 4 P. M. He urinates involuntarily two and three times every afternoon, between 2 and 5. Never in the morning or in the night. He says he has not the slightest desire until he begins to pass stool, and then he cannot control himself. When he does feel an inclination he cannot control himself, but is obliged to go at once. His urine stains his clothes a reddish brown and is very offensive. He says when he has an involuntary stool he has a pain start from the base of the spinal column and run up his back to the brain, in the top of his head, and remain there for an hour. He almost always urinates with his stools, and only has the above pain when the stool alone occurs."

The peculiar pain running up the back is a symptom characteristic of Phos., and as that is the most peculiar symptom it was taken as the guiding symptom of the case. (See *Gregg's Illustrated Repertory.*) "*Darting pains, during stool, from the os coccygis through the spine as far as the vertex, the head being drawn backward by it,*" page 77, plate 5. Phos. also has paralysis of the sphincter ani. (Bell., Gels., Hyos., Graph., and others.) Phos. has a brown stool, and it is offensive. It has also aggravation from excitement and fright. Looking over the first symptoms, it will be seen that Phos. also has the very prominent symptoms with many others, the involuntary stool and urine. The child takes cold easily, and it settles in the respiratory apparatus, which also strengthens the choice. The P. M. agg. I cannot find under Phos., but so small a condition cannot contraindicate the remedy, in view of the fact that none of the other remedies correspond to the peculiar symptoms so well as Phos. Phos. 5m., one dose at night, cured the case promptly.

